# New Discovery

**Published:** 1859-12

**Authors:** 


New Discovery.—Mr. R. Smith, chemist, of Blackford, has of late discovered a process for converting the brown sugar of lead into the white acetate of lead. The process is said to be easily managed, and it may be anticipated that this process will be of great advantage to mnnufacturers, calico-printers, and others. Nor is this all. There are two new compounds formed during the process of great interest. The one is a beautiful scarlet-colored pigment, and the other is a yellow. These colors are well adapted to all kinds of oil painting, water coloring, and paper painting. We have had an opportunity of seeing the colors, and in our opinion they are very fine. The scarlet is equal to vermilion, while the price of it will not exceed the one-third of that substance.—*Stirling (Scot.) Journal.*

